# Grounding magnitudes

**DOI:** 10.3389/fpsyg.2013.00410

**Published:** 2013-07-08

**Authors:** Luca De Simone

**Affiliations:** Area of Neuroscience, International School for Advanced StudiesTrieste, Italy

Over the last few decades, different studies have been directed toward a better understanding of how humans use perceptual information in order to estimate magnitudes. Converging data from different research domains indicate that the processing of time, space, and other quantities frequently unfold together in the human brain and cognition. The idea of a common metric that regulates the ability to extract and manipulate information about quantities is best described by A Theory Of Magnitude (ATOM; Walsh, 2003; Bueti and Walsh, 2009). ATOM considers space, time, and numbers as represented in a generalized magnitude system based on a common code that is essential to implement bodily actions. The underlying overlap in the neuro-cognitive processing of magnitudes is mediated by a brain system that primarily involves the parietal and the prefrontal cortex (Bueti and Walsh, 2009; Vicario and Martino, 2010). On the behavioral level, evidence like the Spatial Numerical Association of Response Codes (SNARC effect; Dehaene et al., 1993), show that numerical values are automatically mapped into space. Similarly to numerals, perceptual attributes like size and luminance are accessed faster when “less” is on the left, and when “more” is on the right (Ren et al., 2011). Exposure to numbers can bias visual line bisection (de Hevia et al., 2006), and shift attention in the space (Fischer et al., 2003). Short and long time intervals in the millisecond to seconds range are also represented spatially, respectively from left to right (Ishihara et al., 2008; Vallesi et al., 2008; Vicario et al., 2008; Di Bono et al., 2012). In addition, a variety of findings has highlighted the commonality in the time-quantity dimension: The effect of dual task paradigms (Brown, 1997), the similarity between time and numerical sensitivity (Roitman et al., 2007), the time-number contrast in stroop-like tasks (Dormal et al., 2006), and the perceptual interference of quantity on temporal judgment (Oliveri et al., 2008; Vicario et al., 2011), all suggest a tight link between temporal and numerical cognition.

More recently, researchers have begun to study the approximation of quantities considering resources that are distributed across the brain, the body, and the environment. This approach extends the notion of embodied cognition (e.g., Barsalou, 1999; Wilson, 2002) to the study of magnitude estimation. Like other forms of knowledge, human ability to ponder and weigh quantities may be grounded in sensorimotor representations and in their simulations. It has been shown, for example, that body posture can influence magnitude estimation (Eerland et al., 2011), and that lateral head (Loetscher et al., 2008) or eye turns (Loetscher et al., 2010) affects the generation of random numbers. Furthermore, tactile information interacts with numerical cognition showing cross-modal effects (Krause et al., 2013), and irrelevant magnitude information such as weight interferes with the SNARC (Holmes and Lourenco, 2013). Moreover, considerable evidence supports the notion that processing numerals involves the activation of motor representations (Sato et al., 2007). For example, perceiving numbers affects the planning of hand actions (Badets et al., 2007; Lindemann et al., 2007; Andres et al., 2008), and the execution of random finger movements (Daar and Pratt, 2008; Vicario, 2012). Besides, time processing appears to be biased by the spatial position of the stimuli in the environment (Vicario et al., 2008, 2009), by the manipulation of the observer's egocentric reference frame (Vicario et al., 2011), and by the perception of implied body actions (Nather et al., 2011).

This line of research promises to reveal important features of human magnitude cognition especially if the brain, the body, and the environment are not considered to be linked in mechanistic terms. Therefore, it may be appropriate to study the agent, his body, and his environment as coupled in a dynamic system (Beer, 2000; Juarrero, 2002). In this view, a promising avenue of investigation aims to highlight transcultural and individual differences in spatial, temporal, and numerical cognition. Indeed, if situated actions underlie cognitive processes (Varela et al., 1991; Barsalou, 2009), exploring culturally-mediated and individual differences in magnitude estimation will be highly informative. For example, finger counting habits in different cultures have been found to be associated with different speed numerical values are accessed (Domahs et al., 2010), while individual learning abilities for temporal tasks in the millisecond range are correlated with functional and structural changes of the brain sensorimotor areas (Bueti et al., 2012). Notably, patients affected by specific sensorimotor deficits such as focal hand dystonia (Abbruzzese et al., 2001), demonstrate impaired prediction of the temporal duration of body movements (Avanzino et al., 2013).

It should be noted, as also stated in ATOM, that the reciprocal mapping of space, time, and quantity is used for action. However, little is known about how the development of higher forms of motor control influences the common representation of magnitudes. Nine months old infants generalize learning about size, numerosity, and time bi-directionally (Lourenco and Longo, 2010), demonstrating an early co-representation of magnitudes during development. As proposed by Hommel and Elsner (2009), infants learn to perform goal-directed actions through contingencies between self-performed movements and their expected effects. Thus, learning to represent the relevant actions to be performed may be critical also to generalizations about regularities of quantities in the physical world. Associations between actions and their sensory consequences may be used to map together quantitative aspects of space and time in sensorimotor representations.
